# Knowledge and practices on anaemia among women of reproductive age in India

**DOI:** 10.6026/973206300212799

**Published:** 2025-08-31

**Authors:** Meena Samant, Amita Sinha, Divya Suman, Amrita Saxena, Bhavika Chauhan, Kanchan Rani

**Affiliations:** 1 Department Obstetrics and Gynaecology, Kurji Holy Family Hospital, Patna, Bihar, India; 2 Department of Community Medicine, Nalanda Medical College & Hospital, Patna, Bihar, India; 3 Department Obstetrics & Gynaecology, Jannayak Karpoori Thakur Medical College & Hospital, Madhepura, Bihar, India; 4 Department of Obstetrics and Gynaecology, Baba Raghav Das Medical College, Gorakhpur, Uttar Pradesh, India; 5 Department of Obstetrics and Gynaecology, Shreyas Medicare M.N. Mehta Hospital, Vapi, Gujarat, India; 6 Department of Obstetrics and Gynaecology, Teerthanker Mahaveer Medical College and Research Centre, Moradabad, Uttar Pradesh, India

**Keywords:** Anaemia, KAP survey, reproductive-age women, iron-deficiency, heavy menstrual bleeding, oral iron supplements, public health awareness

## Abstract

Anaemia is a major public health challenge in India, with NFHS-5 reporting over 57% prevalence among reproductive-age women. Therefore, it is of interest to assess knowledge, attitudes and practices (KAP) regarding anaemia among 1,460 women from six Indian states using a structured FOGSI questionnaire. While most participants recognized anaemia as a blood deficiency, misconceptions about its causes, symptoms and prevention persisted. Although 79.8% supported iron supplementation, only 29.5% reported regular treatment practices and awareness of dietary and micronutrient factors remained low. Comprehensive community-based education beyond iron tablets is crucial to bridge the knowledge-practice gap and reduce anaemia burden.

## Background:

Anaemia, particularly iron deficiency anaemia (IDA), remains a significant public health concern in developing countries, disproportionately affecting women of reproductive age. The World Health Organization (WHO) estimates that approximately 30% of non-pregnant women and 41.8% of pregnant women globally are anaemic, with the burden substantially higher in South Asia [1]. In India, the National Family Health Survey-5 (NFHS-5, 2019-2021) reported that more than 57% of women aged 15-49 years were anaemic, reflecting a persistent gap despite multiple government initiatives [2]. Anaemia in reproductive-age women arises from diverse causes including inadequate dietary intake, menstrual blood loss, short inter-conceptional periods, multiple births, parasitic infections and impaired iron absorption [3]. Although oral iron supplementation is widely promoted, many women perceive iron tablets as the sole preventive measure, which limits broader compliance and engagement with sustainable dietary and behavioural changes [4]. Heavy menstrual bleeding, an underreported gynaecological issue, is another important yet neglected contributor to iron deficiency [5]. Awareness of such conditions, along with dietary counselling, is critical for the success of government programmes. Knowledge, Attitude and Practice (KAP) studies provide essential insights into community understanding of anaemia and can guide public health interventions and educational strategies [6]. Despite large-scale initiatives like Anaemia Mukt Bharat, Weekly Iron and Folic Acid Supplementation (WIFS) and deworming campaigns, gaps persist in actual knowledge and practices, especially in rural and semi-urban populations [7]. Anaemia not only compromises women's health but also affects cognitive performance, productivity, quality of life and maternal-foetal outcomes. In pregnancy, untreated anaemia increases the risk of preterm birth, low birth weight, postpartum haemorrhage and maternal mortality, imposing both health and economic burdens [8]. Furthermore, healthcare communication often fails to highlight other contributors such as vitamin B12 deficiency, gastrointestinal malabsorption and menstrual disorders like menorrhagia, which also perpetuate anaemia [9]. Hence, comprehensive educational strategies that extend beyond iron supplementation are urgently required. Bihar and north-central India represent socio-demographically diverse regions where anaemia prevalence is presumed high but understudied, whereas southern and western India report comparatively better health outcomes [10]. Therefore, it is of interest to assess the knowledge and practices regarding anaemia among reproductive-age women and to identify misconceptions and barriers to effective prevention and treatment.

## Materials and Methods:

A cross-sectional observational study was conducted to assess the knowledge and practices related to anemia among women of reproductive age (15-49 years) in Bihar, Jharkhand, Uttar Pradesh, Madhya Pradesh and Gujarat and Telangana. The study was carried out over a three-month period of April 2019 to July 2019 and employed a structured questionnaire-based approach to collect data from participants. The questionnaire was adapted from the format developed by the FOGSI Clinical Research Committee (2019-2022) and consisted of 11 close-ended questions. The first five questions assessed knowledge related to the definition, causes, symptoms and prevention of anemia. The subsequent five questions focused on individual practices, such as use of iron supplements, dietary habits and response to anemia-related symptoms. The final question sought participants' willingness to contribute further to awareness and prevention programs. A total of 1,460 women were selected through random sampling across various districts of Bihar including Madhepura, Patna, Purnea, Sasaram, Motihari, Lakhisarai, Danapur, Maner and Ranchi (Jharkhand), Vapi (Gujarat), Bhilai (chhattisgarh), Moradabad & Gorakhpur (Uttar Pradesh) and Telangana to ensure demographic, geographic and cultural diversity. Eligibility criteria included women aged 15-49 years who gave consent to participate in the survey. Data were collected by trained field workers and doctors using printed forms and later entered into SPSS software (version 25) for statistical analysis. Descriptive statistics including frequencies and percentages were used to summarize the responses. Cross-tabulations were conducted to explore associations between geographic locations and response patterns. Chi-square tests were applied to determine statistical significance of associations between knowledge and practice items across districts, with a p-value < 0.05 considered statistically significant.

## Results:

A total of 1,460 reproductive-age women participated in the survey. The highest number of respondents were from Bhilai (13.4%), followed by Purnea (13.0%) and Vapi, Gujarat (9.8%). The distribution of participants across locations is shown in Table 1 (see PDF). In response to Q1 (definition of anemia), 74.5% (n=1088) correctly identified it as a reduction in blood levels, while 18.6% (n=271) believed anemia is due to low hemoglobin only (Table 2 - see PDF). For Q2, 41.6% (n=608) identified fatigue and dizziness as symptoms, whereas only 20.2% (n=295) associated it with paleness. When asked about the cause of anemia in Q3, 32.2% (n=470) correctly linked it to nutritional deficiency, while 18.5% (n=270) believed it could result from other factors such as infections or hereditary causes. A large proportion of participants (79.8%) reported taking iron tablets during pregnancy (Q6). Q7 revealed that 74.9% (n=1094) considered anemia to be a disease. However, actual treatment-seeking behavior (Q9) was suboptimal, with only 29.5% (n=431) regularly treating anemia and 31.8% (n=464) stating they do nothing. Concerningly, only 9.6% (n=140) cited regular medical consultation for anemia (Table 3see PDF).

## Discussion:

The results of our study reflect a considerable awareness of the term "anaemia," with 74.5% of participants correctly identifying it as a deficiency in blood. However, deeper understanding regarding the etiology, complications and holistic management of anaemia appears limited. These findings are consistent with other studies conducted in low- and middle-income regions where surface-level awareness does not translate into actionable health behaviour [1, 2]. Although over half of the participants recognized iron deficiency as a major cause of anaemia (56.5%), a significant proportion failed to link other contributors such as heavy menstrual bleeding, poor absorption, or folate and vitamin B12 deficiencies to the condition. Prior researches highlight the complexity of anaemia, emphasizing the need for education beyond iron supplementation alone [3, 4]. Additionally, only 41.6% associated anaemia with fatigue and dizziness, suggesting that symptom recognition is another gap that can hinder timely diagnosis and intervention [5]. Practice-based responses reflected encouraging trends in some areas. A large majority (79.8%) reported taking iron tablets during pregnancy and 84.4% women stated they consulted doctors when symptomatic. These figures are notably higher than those reported in similar community-based studies in northern India and sub-Saharan Africa, where access to antenatal care and health-seeking behaviour is more limited [6, 7- 8]. However, the discordance between awareness and sustained practices was evident, with only 29.5% undergoing regular treatment for anaemia and 31.8% taking no action at all, indicating a substantial compliance gap [9].

The misconception that anaemia is not a serious condition remains prevalent. While 74.9% considered it a disease, only 81.8% acknowledged its potentially life-threatening consequences. A similar pattern was noted in a cross-sectional study conducted among adolescent girls in Tamil Nadu, where respondents underestimated the health risks associated with chronic anaemia [10]. This underestimation of severity can contribute to neglect in early stages and increase the likelihood of complications in pregnancy and general health. Socio-cultural beliefs and local practices also play a role in shaping attitudes toward anaemia. In the current study, regional variations in knowledge and practices were statistically significant, with districts such as Sasaram and Purnea showing higher awareness and treatment compliance compared to others like Madhepura and Maner. This aligns with the findings of regional studies that suggest urban or semi-urban populations often have better exposure to health information due to education and accessibility [11, 12]. Another crucial aspect is the lack of understanding surrounding heavy menstrual bleeding as a contributing factor.

This topic remains stigmatized and under-discussed in most parts of India. Previous studies have indicated that menstrual disorders are a significant, yet neglected, contributor to iron-deficiency anaemia in women [13]. Thus, any anaemia-related intervention must integrate menstrual health education to enhance impact. KAP surveys such as this provide valuable insights for designing public health interventions. The high willingness (99.9%) of participants to spread awareness about anaemia is a promising indicator for community-based health promotion programs. Similar participatory approaches have shown effectiveness in anaemia reduction initiatives in rural Bangladesh and Nepal [14, 15]. The exceptionally high anaemia prevalence underscores the need for integrated interventions-bridging nutrition education with socioeconomic support-to effectively reduce anaemia rates in this population [16]. Despite the positive attitudes toward health education, challenges such as low literacy, poverty and poor access to fortified foods still impede anaemia prevention in rural India. Multi-pronged interventions involving nutritional supplementation, menstrual health management and behaviour change communication are necessary to address the complex socio-medical factors related to anaemia.

## Conclusion:

Although awareness of anaemia is widespread among reproductive-age women, persistent misconceptions, poor symptom recognition and low adherence to iron supplementation remain major barriers to effective control. These gaps undermine the impact of existing government programmes and delay timely health-seeking behaviour. Strengthening women-centric education, integrating menstrual and nutritional counselling and implementing community-based interventions are crucial to bridge the knowledge-practice gap and reduce the burden of anaemia in India.
